# Prevalence of intestinal helminths and associated factors among school children of Medebay Zana wereda; North Western Tigray, Ethiopia 2017

**DOI:** 10.1186/s13104-018-3556-6

**Published:** 2018-07-04

**Authors:** Tsega Teshale, Shewaye Belay, Desalegn Tadesse, Abraham Awala, Girmay Teklay

**Affiliations:** 1 Department of Laboratory, Aksum University, Po Box: 298, Aksum, Tigray Ethiopia; 2 Department of Pediatrics and Child Health Nursing, School of Nursing, Aksum University, Aksum, Tigray Ethiopia; 3 Department of Parasitology and Entomology, Mekelle University, Mekelle, Tigray Ethiopia

**Keywords:** Helminths, STH, Schistosoma, Schistosomiasis, Mother’s education, Open defecation

## Abstract

**Objective:**

To assess the prevalence of intestinal helminth infections and associated factors among primary school children of Medebay Zana wereda, a northwestern zone of Tigray, northern Ethiopia from March to April 2017.

**Result:**

The prevalence of intestinal helminths was 12.7%. The highest prevalence of intestinal helminth infections was observed in the age group of 11–14 years old and the most prevalent helminths species were *Schistosoma mansoni*. Mothers’ level of education [AOR = 0.27 [0.13–0.58]], place of defecation [AOR = 2.63, 95% CI 1.14–6.02]], hand wash before meals [AOR = 9.0, 95% CI 3.72–21.74]], hand wash after defecation [AOR = 5.77 [1.78–18.63]] and eating unwashed vegetables [AOR = 5.67 [2.19–14.73]] were associated with higher risk of having intestinal helminths detected in stool. In the study area the risk of detecting intestinal helminths in their stool were more associated the improper personal hygiene of the children.

## Introduction

About 610 million school age children of the world are at risk of infection with a common intestinal helminths [1] like *hookworm*, *A. lumbricoides*, *T. trichiura*, *Strongyloides stercoralis*, *Hymenolepis nana* and *Schistosoma mansoni* [2, 3]. Those infections are resulted from socioeconomic factors, cultural practices and poor sanitation which are transmitted by ingestion of soil contaminated egg, poor hand hygiene, eating unwashed fruit, under cooked infected meat and poorly washed vegetable [4]. Skin penetration while walking bare foot on larvae infected soil and skin penetration by swimming in cercariae bearing water are anther transmission mechanism of helminths [5, 6].

Infestation by intestinal helminths is the major public health problem which causes chronic inflammatory disorder such as chronic anemia, growth stunting, protein-calorie malnutrition, fatigue, poor cognitive performance, reduce long term survival, diminished physical fitness and school attendance in school-age children [7, 8]. Intestinal helminths infections like acute and chronic complications of *S. mansoni* also cause very severe consequences like abdominal pain, diarrhea, hepatosplenomegaly, portal hypertension, and upper gastrointestinal bleeding [9].

Worldwide, about 100 million of intestinal worm harboring school-children experience stunting or wasting [10, 11]. Severity in intestinal helminths resulted from fluctuation in metabolic rate, anorexia, chronic anemia, obstructing intestinal lumen of children associated with heavy worm-load [12].

In Ethiopia, intestinal helminths are resulted from poor environmental sanitation, unsafe human waste disposal systems, lack of safe water supply and low socio-economic status of the country [13–18]. The main reason for their persistent presence everywhere is due to frequently low rank in the list of priorities in public health programs because the effect of helminthic infections are not directly measured explicitly in terms of mortality figures [19].

Though studies on intestinal helminths and associated factors have been conducted in different parts of Ethiopia [10, 20–24], the prevalence of intestinal infection in the local area has not yet properly documented. This study will help for school community to improve their environmental sanitation and to health office of Medebay Zana wereda to design periodic mass-deworming program. Therefore, the aim of this study was to assess the prevalence of intestinal helminths and associated factors among School Children of Medebay Zana wereda; Tigray, Ethiopia.

## Main text

### Study design and setting

A school based cross-sectional study design was used to collect data in eight primary schools of Medebay Zana wereda from March to April 2017, located at 282 km from Mekelle and about 1060 km far from Addis Ababa. Its geographical location is between 38°20′E longitude and 14°06′N latitudes [25]. About 9% of all houses in urban had access to safe drinking water and 4% of rural houses had toilet facilities [26].

### Sample size calculation

The sample size was calculated using single population proportion formula by taking the highest sample giving from both prevalence and associated factors. We using the previous prevalence from a study done in Enderta [10] i.e. 41.46% with 5% margin of error and 95% confidence interval. The final sample size with 10% contingency was 410.

### Sampling technique

From the total primary schools in the wereda, eight schools were selected randomly four from rural and four from urban areas. From the eight selected schools with 1–8 grades, 410 children were taken by using simple random sampling technique. Kato-katz technique were also use for the laboratory investigation.

### Source and study population

All students in elementary schools of Medebay Zana wereda were considered as source population and all randomly selected students grade 1–8 in eight selected elementary schools were considered as our study subjects.

### Data collection procedure and instruments

The data was collected using interviewer administered structured questionnaire adapted from study done in Lumam town, north west Ethiopia [27] and stool sample. The adapted questionnaire was modified and contextualized to fit the local situation and research objective. After obtaining oral consent from teachers and parents and assent from the students, each student was given a coded stool collection container (clean plastic cup) and applicator sticks and instructed to bring 6 g of his/her own fresh stool. Two BSc in graduates’ in laboratory technology were recruited as data collectors and one senior professional nurse for screening of complications. Stool collection was supervised throughout the time. On delivery of the stool specimen the code of parasitological investigation was filled properly on the prepared parasitological format that was attached with questionnaire.

Following stool examination using Kato-Katz technique, children who become positive for *S. mansoni* infection were checked for palpable splenomegaly and hepatomegaly. Those found with splenomegaly and hepatomegaly were informed to their parents to take them to hospital for further checkup and for free care of their complications.

### Study variables

The dependent variable was detection of helminths in stool samples and the independent variables were socio-demographic factors like: age, sex, fathers and mothers level of education, residence and other associated factors like:- shoes wearing habit, place of defecation, water source, hand washing habit before meal, hand washing habit after defecation and habit of eating improperly washed raw vegetables. Those variables was asked to the caretaker of the children.

#### Data quality assurance

Training was given for data collectors. The questionnaire was prepared originally in English and then translated into the local language of study area (Tigrigna). Questionnaires were pretested on 5% of the sample size at Mekane-Eyesus primary school. All reagents were checked for expired date. Close supervision was done by the principal investigator.

#### Data processing and analysis

The Data was coded, entered, cleaned, edited and analyzed using SPSS ver.22. The prevalence of intestinal helminths was reported in percentages of detected helminths in stool of the children. Binary logistic regression was used to examine the association between the dependent variable and each independent variable. Variables which showed statistical significance during bivariate analysis at p value ≤ 0. 20 were entered to multivariate logistic regression to isolate an independent effect of the predictors. Adjusted odds ratios (AOR) with 95% CI, were estimated to assess the strength of associations and statistical significance was declared at a p-value < 0.05.

### Results

#### Socio-demographic characteristics of the study subjects

A total of 410 school age children were examined for intestinal helminths with stool sampling success rate of 100%. Out of these respondents 58.5% were males and 238 (58%) were live in the rural area. About 238 (48.8%) of the participants were belonged to households with a family size range from 4 to 6. The mean age of study participants was 11.41 ± 2.202 ranging 9–19 years old (Table 1).

**Table 1:** Socio-demographic characteristics with respect to their prevalence of intestinal helminths of wereda Medebay Zana, Tigray, Ethiopia, 2017

Socio-demographic characteristics	Intestinal helminths		Total
	Positive (%)	Negative (%)	
Age			
6–10	11 (12.5)	77 (87.5)	88
11–14	36 (13.5)	230 (86.5)	266
15–19	5 (8.9)	51 (91.1)	56
Gender			
Male	27 (11.2)	213 (88.8)	240
Female	25 (14.5)	145 (85.3)	170
Religion			
Orthodox	49 (13)	340 (87)	389
Muslim	3 (14.3)	18 (85.7)	21
Residence			
Urban	30 (17.4)	142 (82.6)	172
Rural	22 (22)	216 (90.8)	238
Family size			
1–3	3 (11.1)	24 (88.9)	27
4–6	31 (15.5)	169 (84.5)	200
> 6	18 (9.8)	165 (90.2)	183
Mother education			
Illiterate	21 (9.2)	208 (90.8)	229
Literate	31 (17.1)	150 (41.9)	181
Father’s education			
Illiterate	31 (14.7)	108 (85.3)	211
Literate	21 (10.6)	179 (89.4)	199
Use river water			
Yes	39 (9.6%)	366 (90.4%)	405
No	2 (40%)	3 (60%)	5
Purpose to use river water			
To drink wash and swim	4 (9.8%)	37 (90.2%)	41
Only to wash and swim	37 (10%)	332 (90%)	369
Shoes wearing			
Yes	26 (8%)	301 (92%)	327
No	15 (18%)	68 (82%)	83
Cross flowing river and irrigational area			
Yes	32 (12.5%)	225 (87.5%)	257
No	9 (5.9%)	144 (94.1%)	153

#### Prevalence of intestinal helminths

In this study 12.7% (CI 9.60–16.30) of the participants have at least one intestinal helminths detected in their stool. The detected helminths in stool was high in the age range of 11–14 years old with prevalence of 36 (13.5%). In the study 405 (98.8%) of the children uses river water. Among them 39 (9.6%) were had intestinal helminths and 4 (9.8%) of them uses the river water for drinking, washing and swimming. About 15 (18%) of the children did not wear shoes and 32 (12.5%) were cross the flowing river and irrigational area in their daily activity (Table 1).

#### Distribution of single and double infection

Five different helminthic species were recorded (*A. lumbricoides*, Hookworm, *S. mansoni*, *H. nana* and *T. trichiura*). Out of the detected helminths infection 33 (63.5%) were occur in the age range of 11–14 years. From the total intestinal helminths detected, 49 (94.2%) of the children were having single infection of the helminths and 3 (5.8%) were co-infected with two different helminths *A. lumbricoides* and *T. trichiura* (Table 2).

**Table 2:** Distribution of single and double infection of wereda Medebay Zana, Tigray, Ethiopia, 2017

Variables N = 52	Single infection					Double infection	Total (%)
	*S. mansoni*	*A. lumbricoides*	Hookworm	*H. nana*	Total n (%)	*A. lumbricoides and T. trichiura*	
Sex							
Male	12 (23%)	4 (7.7%)	6 (11.5%)	3 (5.8%)	25 (48%)	2 (3.8%)	2 (3.8%)
Female	8 (15.4%)	2 (3.8%)	11 (21.2%)	3 (5.8%)	24 (46%)	1 (1.9%)	1 (1.9)
Total	20 (38.5%)	6 (11.5%)	17 (32.7%)	6 (11.5%)	49 (94%)	3 (5.8%)	3 (5.8)
Age							
6–10	4 (7.7%)	2 (3.8%)	4 (7.7%)	1 (1.9%)	11 (21.2%)	0	0
11–14	14 (27%)	4 (7.7%)	11 (21.2%)	4 (7.7%)	33 (63.5%)	3 (5.8%)	3 (5.8%)
15–19	2 (3.8%)	0	2 (3.8%)	1 (1.9%)	5 (9.6%)	0	0
Total	20 (38.5%)	6 (11.5%)	17 (32.7%)	6 (11.5%)	49 (94%)	3 (5.8%)	3 (5.8%)
Residence							
Urban	13 (25%)	4 (7.7%)	9 (17.3%)	1 (1.9%)	27 (51.9%)	0	0
Rural	7 (13.5%)	2 (3.8%)	8 (15.4%)	5 (9.6%)	22 (42.3%)	3 (5.8%)	3 (5.8%)
Total	20 (38.5%)	6 (11.5%)	17 (32.7%)	6 (11.5%)	49 (94.2%)	3 (5.8%)	

#### Splenomegaly and hepatomegaly with *S. mansoni*

In this study, the most predominant helminthic infection was *S. mansoni* 20 (38.5%). From all children infected with *S. mansoni*, 5% were with splenomegaly and 25% had hepatomegaly and were linked to hospital for free care.

#### Risk factors for intestinal helminths infections

In the bivariate logistic regression at p-value of ≤ 0.2, residence, mother’s educational status, place of defecation, hand washing before meal, hands washing after defecation, shoes wearing habit and eating unwashed vegetable were statistically significant with having intestinal helminths infections in the stool sample.

After entering these variables to multivariate analysis by using backward elimination method, Mother’s educational status, place of defecation, hand washing before meal, hands washing after deification and eating unwashed vegetable were statistically significant with the detected intestinal helminthic infections in the stool at p-value < 0.05 (Table 3).

**Table 3:** Risk factors of intestinal helminth infections of wereda Medebay Zana north western Tigray, Ethiopia, 2017

Variables	Laboratory result		CO (CI 95%)	AOR (CI 95%)	p-value
	Positive n (%)	Negative n (%)			
Level of education of mother					
Illiterate	21 (9.2)	208 (90.8)	0.489 [0.270–0.883]	0.272 [0.127, 0.580]	0.001*
Literate	31 (17.1)	150 (82.9)	1	1	
Place of defecation					
Latrine	9 (6)	142 (94)	1	1	0.023*
Open field	43 (16.6)	216 (83.3)	3.329 [1.574–7.037]	2.626 [1.145, 6.020]	
Hand washing before meal					
Yes	10 (5)	209 (95)	1	1	0.000*
No	42 (22)	149 (78)	5.959 [2.898, 12.25]	8.999 [3.725, 21.74]	
Hand washing after defecation					
Always	5 (9)	80 (94.1)	1	1	0.003*
Sometimes	19 (9)	192 (91)	1.583 [0. 571–4.387]	1.536 [0.463, 5.094]	
Not at all	28 (24.6)	86 (75.4)	5.209 [1.918, 14.14]	5.766 [1.784, 18.63]	
Eating unwashed vegetable					
Always	24 (28)	61 (71. 8)	5.587 [2.519, 12.38]	5.675 [2.186–14.73	0.0001*
Sometimes	18 (10.4)	155 (89.6)	1.649 [0.7373.692]	1.139 [0.442–2.932]	
Not at all	10 (6.6)	142 (93.4)	1	1	

### Discussion

The overall prevalence of intestinal helminths recorded in this study was 12.7%. This was almost comparable with the study done in Were-abay 12.22% [16] and Babile town 13.8% [17]. But the result of this study was much lower than the findings reported in Adwa town 69% [28], Lumame town 54%) [27], Zegie Peninsula 69.1% [29] and in northern Gondar 66.7% [30]. This could be due to the topographic and study period difference in which the communities would improve their living standard, personal and environmental hygiene through time.

*Schistosoma mansoni* was the most predominant intestinal helminthes with prevalence of 20 (38.5%) and 4.9% from the total participant. This is higher than the study done in Were-Abaye with less than one percent [16]. This might be due to the presence of irrigational areas and flowing river that favor breeding of the intermediate host (snail) in which the infected snails shed cercariae into the water and penetrate the intact human skin. The second intestinal helminth detected was Hookworm with prevalence of 4.14% from the total participant and 17 (32.7%) from the detected helminths in stool. This was higher than a study done in Chencha town 2.2% [31] and lower than studies conducted in Babile town 6.7% [32] and Jimma town 12.9% [33]. The prevalence of *H. nana* in this study was 1.46% from the total participant and 6 (11.5%) from the total helminths detected.

Out of the total helminths detected, 49 (94.2%) were infected with single helminth infection. Lower than study done in Chencha town 19% [31]. Unlike the studies done in in Adwa town [29] with double infection of 18.4%, in our study area only 3 (6%) of the school children were infected with two different helminths (*A. lumbricoides* and *T. trichiura*).

The parents of children at high level of education provides better environment for their children. In contrast to this idea, in our study the odds of intestinal helminths in children with illiterate mothers were 72.8% less likely to be infected than children with literate mothers at p = 0.001. This may be due to the similarity of the environmental condition between the schools and living environment.

Human contaminative activities such as open field defecation and use of unhygienic water supply for bathing and drinking favor transmission of intestinal helminths. In our study, students who excrete in open field were about three (p = 0.023) times more likely to have intestinal helminths in their stool than those who defecate in latrine. This was different from the study done in Lumame town [27]. This difference might be due to the difference in topological and environmental sanitation between the two study areas. Students who didn’t wash their hands before meal were about nine (p = 0.000) times more prone to develop intestinal helminths than those who wash their hands before meal. The helminths might be confined into the nail of the child and may cause infections as they ingest food.

The odds of having intestinal helminths among children who eat unwashed or undercooked vegetables in the stool of the children were about six times higher (p = 0.000) than those who eat washed or cooked vegetables. This was similar with the study done in Lumame town [27]. This might be due to the unwashed or undercooked vegetables and fruits may create a favorable media to eggs of these helminths to infect children.

### Conclusion

The overall prevalence of intestinal helminths recorded in this study was 12.7%. Therefore, Health education programs such as proper personal hygiene should be strengthened in the school and community.

## Limitations

The study did not identify the actual cause of hepatosplenomegaly in *S. mansoni* positive children. The absence of broader health status of the community was another limitation in which environmental assessment was not conducted around the schools and communities. The study also shares the limitation of the cross-sectional study design.

## Abbreviations

AOR: adjusted odd ratio
COR: crudes odd ratio
SPSS: Statistics Package for Social Science
STH: soil transmitted helminths
BSc: Bachelor of Science
